# Acute Splenic Artery Thrombosis and Infarction Associated with COVID-19 Disease

**DOI:** 10.1155/2020/8880143

**Published:** 2020-09-04

**Authors:** Osama Qasim Agha, Ryan Berryman

**Affiliations:** ^1^Department of Internal Medicine, Creighton University School of Medicine, Phoenix, Arizona, USA; ^2^Department of Internal Medicine, St Joseph's Hospital and Medical Center, Phoenix, Arizona, USA; ^3^Department of Internal Medicine, University of Arizona College of Medicine, Phoenix, Arizona, USA

## Abstract

Coronavirus 2019 disease (COVID-19) is a viral illness caused by severe acute respiratory syndrome coronavirus 2 (SARS-CoV-2). It emerged in Wuhan, China, in December 2019 and has caused a widespread global pandemic. The symptoms of COVID-19 can vary from mild upper respiratory symptoms to severe pneumonia with hypoxemic respiratory failure. Multiple studies and reports have reported a hypercoagulable state associated with this disease, and various recommendations have emerged to guide the use of anticoagulants for prophylaxis. We are reporting a case of symptomatic acute splenic thrombosis causing splenic infarction in a patient suffering from a severe case of COVID-19 and despite the use of an intermediate dose of low-molecular-weight heparin (LMWH). The patient was treated with full-dose anticoagulation and was eventually discharged home on a direct oral anticoagulant.

## 1. Introduction

Approximately 21 million individuals have been diagnosed with COVID-19 worldwide, and approximately 761 thousand fatalities have been reported as of August 21, 2020 [1]. Hypercoagulability leading to venous thromboembolism (VTE) and arterial thrombosis has been reported in multiple studies, and various recommendations to prevent these events have emerged. Multiple ongoing studies are still evaluating the most appropriate dosing to prevent VTE.

## 2. Case Presentation

A male patient in his 60s presented to the emergency department with worsening dyspnea for two weeks. It was associated with fevers, cough, and diarrhea. He tested positive for COVID-19 one week prior to presentation, and he was started on hydroxychloroquine 400 mg daily by his primary care physician, but his symptoms continued to progress. He had a history of asthma, obstructive sleep apnea, morbid obesity, immunoglobulin G (IgG) deficiency, and hypertension. His home medications included amlodipine, olmesartan, Hizentra, and the recently prescribed hydroxychloroquine. The patient was a nonsmoker, and he drank alcohol only occasionally. His vital signs included a temperature of 39.5°C, a respiratory rate of 20-24, an oxygen saturation of 90% on room air, and a body mass index of 54 kg/m^2^. He was in no distress, and his lungs were clear on auscultation. The rest of the physical exam was normal. The patient was initially admitted to a telemetry unit but was then transferred to the intensive care unit due to progressive hypoxia requiring high flow oxygen. On day 7 of admission, he complained of moderate, dull, and left-sided abdominal pain that required IV morphine. His abdomen was soft, nondistended, and nontender, and he had no organomegaly on palpation.

**Figure 1:**
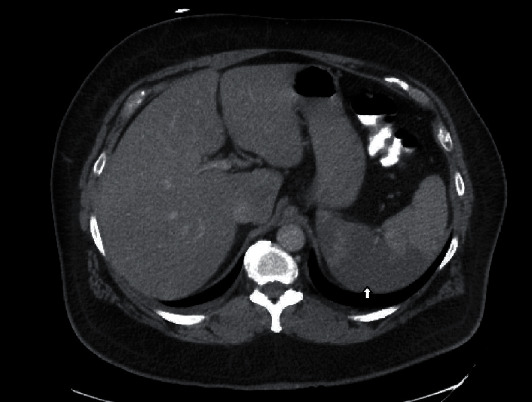
An area of hypodensity involving the right half of the spleen consistent with splenic infarction (arrow).

**Figure 2:**
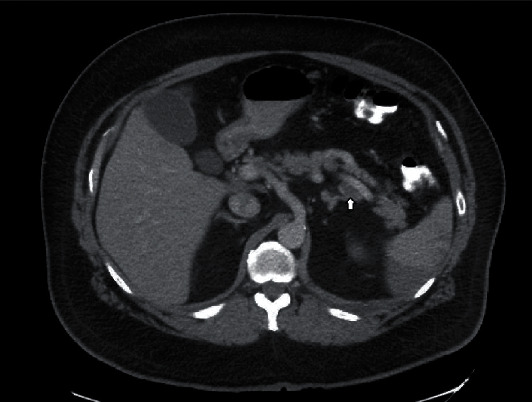
A filling defect within the mid to distal splenic artery consistent with splenic artery thrombosis, transverse view.

**Figure 3:**
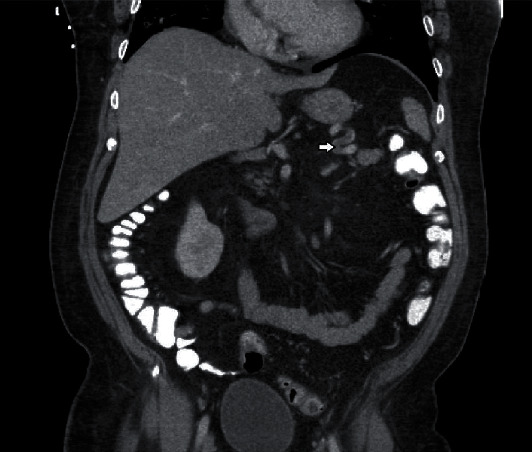
A filling defect within the mid to distal splenic artery consistent with splenic artery thrombosis, coronal view.

On admission, his complete blood count (CBC) and comprehensive metabolic panel (CMP) were unremarkable. However, he had an elevated D-dimer level at 259 ng/ml, CRP level at 86.6 mg/l, ferritin level at 1,472 ng/ml, and procalcitonin level at 0.09 ng/ml. Chest X-ray showed patchy opacities in the right upper and lower lobes. On day 7, his white blood cell count was slightly elevated at 11.2 thousand/*μ*l and CMP remained unremarkable. His repeat D-dimer level was 1,088 ng/ml, and his repeat ferritin level was 3,038 ng/ml. A CT of the abdomen and pelvis with IV and PO contrast showed acute splenic artery thrombosis and infarction of greater than 50% of the splenic volume (Figures 1–3).

The patient was receiving enoxaparin 40 mg twice daily prior to making the diagnosis of splenic infarction. He was then switched to heparin drip for 24 hours and then to enoxaparin 1 mg/kg twice daily. The patient's respiratory status improved, and he was weaned off oxygen. His abdominal pain also improved gradually, and he required less opiates. On day 21, the patient was discharged home on oral rivaroxaban.

## 3. Discussion

Hypercoagulability is defined as an increased risk of thrombosis in veins, arteries, or both due to an acquired or a hereditary disorder [2]. Splenic infarction is a rare disorder that can present with left-sided abdominal pain and can be secondary to a hypercoagulable state [2]. COVID-19 has been reported in multiple studies to be associated with hypercoagulability and an increased risk for venous and arterial thromboembolism. The reported abnormal coagulation parameters include elevated D-dimer, fibrin degradation products (FDP), and platelet count with low antithrombin values [3–5]. Abnormal thromboelastography (TEG) values, including decreased *R* and *K* values and increased values of *K* angle and MA, are also consistent with hypercoagulability [6]. While earlier studies reported that some patients had prolonged activated partial-thromboplastin time (aPTT) and expressed concern for increased bleeding risk, a recent study found that the majority had positive lupus anticoagulant which can prolong aPTT and is associated with an increased risk of thrombosis [7]. Elevated D-dimer and FDP have also been associated with more severe disease and poorer prognosis [5, 8].

Pulmonary embolism has been the most common thrombotic event associated with COVID-19 and sometimes despite the use of prophylactic or therapeutic-dose anticoagulation [9, 10]. Large-vessel ischemic stroke and acute upper or lower limb ischemia have also been reported [11–13]. More recently, abdominal visceral infarctions including renal infarction, splenic infarction, and small bowel infarction have been reported [14, 15].

The use of prophylactic-dose low-molecular-weight heparin (LMWH) has been shown to be associated with lower mortality in patients with severe COVID-19 or D-dimer levels more than 6 times the upper normal limit [16]. Current society guidelines support the use of standard prophylactic-dose anticoagulants in all hospitalized patients with COVID-19 in the absence of a clear contraindication [17–19]. The routine use of intermediate- or full-therapeutic doses of anticoagulation is not strongly supported by current guidelines [19]. In our case, the patient developed splenic infarction despite the use of intermediate-dose LMWH. Our patient had a severe case of COVID-19 and was morbidly obese. Evolving evidence from recent trials and expert opinions supports the use of either an intermediate dose or a weight-based dose of anticoagulants to prevent VTE in obese or morbidly obese patients [20–24]. We suggest that the patient's weight and the severity of COVID-19 should be considered in addition to the most recent guidelines when deciding about anticoagulant dosing. While the definition of severe COVID-19 varies from one report to another, we suggest using the presence of hypoxemic respiratory failure, acute respiratory distress syndrome, multiorgan failure, or shock as clinical indicators of severe COVID-19 [25].

## 4. Conclusion

COVID-19 disease can be associated with thrombosis in unusual sites including the splenic artery. Splenic artery thrombosis and splenic infarction should be considered in patients with COVID-19 who are suffering from left-sided abdominal pain. Low- or intermediate-dose LMWH might not prevent thrombotic events in patients with severe COVID-19 and morbid obesity. Therefore, full-dose or weight-based anticoagulation should be considered in patients with severe COVID-19 and morbid obesity. Following up on most recent clinical guidelines and the results of ongoing trials is highly recommended.

## Data Availability

The data supporting this case report are from previously reported studies and datasets, which have been cited. These studies are available upon request from the corresponding author at osmagha@gmail.com.
